# Special Issue “Immunoanalytical and Bioinformatics Methods in Immunology Research”

**DOI:** 10.3390/ijms25020979

**Published:** 2024-01-12

**Authors:** Anton Popov, Almira Ramanaviciene

**Affiliations:** 1 NanoTechnas—Center of Nanotechnology and Materials Science, Institute of Chemistry, Faculty of Chemistry and Geosciences, Vilnius University, Naugarduko St. 24, LT-03225 Vilnius, Lithuania; anton.popov@chgf.vu.lt; 2 Department of Immunology and Bioelectrochemistry, State Research Institute Centre for Innovative Medicine, LT-08406 Vilnius, Lithuania

To effectively control and prevent diseases on a global scale, it is essential to employ precise, sensitive, selective, and rapid immunoanalytical methods. Additionally, for monitoring biomolecules within the organism and predicting their interactions, bioinformatics methods show great promise. Immunosensors and other immunoanalytical techniques play a crucial role in this context, detecting specific interactions between antigens and antibodies occurring on the transducer surface. These approaches hold significant potential in both biomedical and bioanalytical fields.

The objectives of disease control include reducing the incidence and prevalence of disease, minimizing its spread, and improving health outcomes. Immunology, as a scientific discipline that includes the study of the immune system, goes hand in hand with immunoanalytical and bioinformatics techniques in addressing disease control issues. Immunoanalytical methods are based on the formation of an immune complex between immobilized antibodies and antigens of interest present in the sample. Various immunoanalytical methods such as enzyme-linked immunosorbent assay (ELISA), fluorescence immunoassay (FIA), immunohistochemistry (IHC), and many others have already become routine methods for use in clinical laboratories, and some diagnostics tests are even designed to be used by patients at home, providing convenient ways to monitor their health status. These methods are particularly important in analysing and quantifying immune system components such as antibodies, antigens, cytokines, cells, etc. Moreover, these techniques have already found applications not just in medicine but also in veterinary, food control, and environmental monitoring [1,2]. The particular importance of immunoanalytical and bioinformatics methods in the field of public health control became extremely clear during the COVID-19 pandemic. Their availability and abundance make it possible to rapidly study this viral disease, suggest possible methods of control, and bring vaccination of the population closer. Additionally, scientists have devoted much effort to understanding the differences in the interaction of specific antibodies with SARS-CoV-2 spike protein of wild-type and the variants of concern [3] and to select the best antigens and antibodies to develop immunosensors, rapid lateral flow tests, and other immunoanalytical techniques for COVID-19 confirmation and monitoring [4]. To get more precise quantitative information about antibody flexibility and conformational changes during interaction with immobilized antigens, the advantages of combined spectroscopic ellipsometry and quartz crystal microbalance with dissipation were revealed [5].

Despite the abundance of techniques already on the market, there is a constant search for opportunities to improve the analytical parameters of existing techniques. For instance, the incorporation of nanomaterials into immunosensors and other immunoanalytical techniques is driving progress by enabling more sensitive, rapid, and even multiplexed detection of analytes [6,7]. The modification of the immunosensor surface by nanostructures and their application as a label are possible strategies for fabricating an immunoanalytical system [8]. One of the key factors in the development of nanomaterial-based systems is the proper binding of antibodies to nanomaterials [9,10]. The physical adsorption and covalent binding of antibodies are commonly used immobilization methods; however, site-directed immobilization of native antibodies, for instance, using proteins A and G, provides increased sensitivity [11]. Moreover, the use of reduced antibody fragments may be a possible alternative for the site-directed orientation of antigen-binding sites of antibodies developing sensitive immunosensors and other immunoanalytical methods [12]. The affinity of used antibodies or their fragments towards antigens is highly important for improving the performance of the immunoanalytical system.

Despite the enormous potential of immunoanalytical methods, the beginning of the bioinformatics era has shown a new direction for research in immunology. With Dayhoff and Ledley’s pioneering work on bioinformatics, it became clear that these methods could be applied in many areas [13]. The active development of bioinformatics is evidenced by the increase in the number of articles in this field from 556 in 2000 to more than 17,000 in 2022 (Web of Science Core Collection). For instance, the convergence of bioinformatics and computational intelligence techniques in the field of computational oncology, particularly in the diagnosis, prognosis, and optimization of cancer therapy, has witnessed significant progress. These synergistic approaches have made remarkable progress in unravelling the intricacies of cancer development and progression, as well as in developing effective therapeutic strategies. This integration of computational methods plays a key role in various aspects of cancer research and treatment [14]. Moreover, machine learning and artificial intelligence are opening a new chapter of bioinformatics in immunology research [15,16]. Bioinformatics is now an integral part of the search for new biomarkers, research into new possible drugs, and vaccine development. It also plays an important role in immune response profiling, immunodiagnostics, and antibody engineering [17,18].

This Special Issue of the *International Journal of Molecular Science* entitled “Immunoanalytical and Bioinformatics Methods in Immunology Research” is devoted to the application of optical analytical methods and a variety of bioinformatics techniques to find indicative solutions for improving the performance of immunoanalytical systems and disease control. Surface plasmon resonance spectroscopy was proposed to evaluate the conjugation of antibodies with quantum dots. An increase in conjugation efficiency should favorably affect the sensitivity of such bioconjugate-based immunoanalytical systems. A theoretical study showed a new approach suitable for the sensitive detection of dye-labelled antibodies using total internal reflection ellipsometry (TIRE) [19]. Moreover, the combination of TIRE with quartz crystal microbalance with dissipation was applied for the investigation of SARS-CoV-2 spike protein with specific antibodies and the modeling of this interaction. Such a system provides insight into the interaction mechanism, allows more precise calculation of kinetic parameters, and its use can be extended to other antigen–antibody pairs. Integrated bioinformatics analysis was applied to identify possible biomarkers and risk factors of psoriasis [20].
